# Uniform bacterial genetic diversity along the guts of mice inoculated with human stool

**DOI:** 10.1101/2025.01.28.635365

**Published:** 2025-01-29

**Authors:** Michael Wasney, Leah Briscoe, Richard Wolff, Hans Ghezzi, Carolina Tropini, Nandita Garud

**Affiliations:** 1 University of California, Los Angeles, Human Genetics, Los Angeles, CA,; 2 University of California, Los Angeles, Interdepartmental Program in Bioinformatics, Los Angeles, CA,; 3 University of California, Los Angeles, Ecology and Evolutionary Biology, Los Angeles, CA,; 4 University of British Columbia, Department of Bioinformatics, Vancouver, Canada,; 5 University of British Columbia, Department of Microbiology and Immunology, Vancouver, Canada,; 6 University of British Columbia, School of Biomedical Engineering, Vancouver, Canada,; 7 Canadian Institute for Advanced Research, Humans and the Microbiome Program, Toronto, Canada

## Abstract

Environmental gradients exist throughout the digestive tract, driving spatial variation in the membership and abundance of bacterial species along the gut. However, less is known about the distribution of genetic diversity *within* bacterial species along the gut. Understanding this distribution is important because bacterial genetic variants confer traits important for the functioning of the microbiome and are also known to impart phenotypes to the hosts, including local inflammation along the gut and the ability to digest food. Thus, to be able to understand how the microbiome functions at a mechanistic level, it is essential to understand how genetic diversity is organized along the gut and the ecological and evolutionary processes that give rise to this organization. In this study, we analyzed bacterial genetic diversity of approximately 30 common gut commensals in five regions along the gut lumen in germ-free mice colonized with the same healthy human stool sample. While species membership and abundances varied considerably along the gut, genetic diversity within species was substantially more uniform. Driving this uniformity were similar strain frequencies along the gut, implying that multiple, genetically divergent strains of the same species can coexist within a host without spatially segregating. Additionally, the approximately 60 unique evolutionary adaptations arising within mice tended to sweep throughout the gut, showing little specificity for particular gut regions. Together, our findings show that genetic diversity may be more uniform along the gut than species diversity, which implies that species presence-absence may play a larger role than genetic variation in responding to varied environments along the gut.

## Introduction

The spatial organization of microbiota along the gut plays a critical role in the microbiome’s function^1^. Previous research in humans and mice has established that different regions of the gut, such as the large and small intestines, are characterized by distinct assemblages of bacterial species^2,3^, and that disruption of this organization is associated with disease^4^. Spatial organization in the microbiome is thought to arise from distinct niches imposed by environmental and physiological gradients along the gut^3,5,6^. However, it remains unknown whether such gradients drive similar spatial organization *within* bacterial species, at the level of strains or individual genetic variants carried by those strains. Understanding spatial organization at this level is important because bacterial genetic diversity is responsible for conferring traits important for the proper functioning of the microbiome and is known to impart several host-relevant phenotypes^7^, including ability to digest food^8^, local inflammation in specific regions of the gut^9^, antibiotic resistance^10^, metabolic capabilities^8,11–14^, and pathogen resistance^15^.

**Figure 1.**
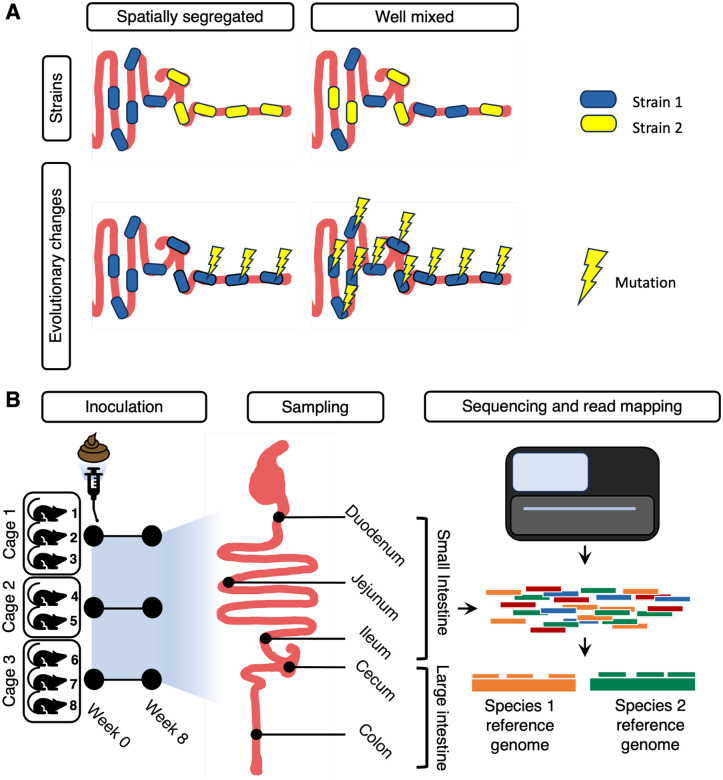
Hypotheses and experimental design. **(A)** Possible organization of genetic diversity along the gut. Strains and their evolutionary changes may be either spatially segregated, or evenly mixed. **(B)** Experimental design. Eight germ-free Swiss Webster mice were inoculated with a single healthy human-derived stool sample. Subsequently, the microbiota was allowed to equilibrate over eight weeks and shotgun sequencing was performed on samples collected along five sites in the gut (small intestine: duodenum, jejunum, and ileum; large intestine: cecum and colon). In our analyses, we refer to duodenum, jejunum, and ileum as regions of the small intestine, and cecum and colon as regions of the large intestine.

There are several ecological and evolutionary mechanisms that could result in either i) spatially segregated or ii) well-mixed genetic variation along the gut (Figure 1A). At an ecological level, spatial segregation could arise from environmental gradients selecting for different strains of the same species along the gut, or from competition between strains. Alternatively, co-colonizing strains may be uniformly distributed along the gut if other, non-spatial niches can sustain within-host strain diversity. A previous study looking at a small number of commensal bacterial species found that different strains can coexist along the gut^16^, but it is unknown how general this phenomenon is, and moreover whether strain frequencies of co-colonizing strains are homogeneous along the gut. At an evolutionary level, spatial segregation of adaptive variants could arise from environmental gradients selecting for different adaptations along the gut. Alternatively, adaptive variants may spread throughout the gut due to rapid migration rates or because they are globally adaptive. Recent work in healthy hosts suggests single nucleotide variants (SNVs) can arise locally along the gut for a few species^16,17^, though these evolutionary changes were not conclusively shown to be adaptive. In another case, a uropathogenic *Enterococcus gallinarum* strain evolved spatially specific adaptive mutations to the mucosa, which caused inflammation^9^. It is unclear how commonly genetic variation segregates along the gut, particularly along the longitudinal axis of the gastrointestinal (GI) tract (i.e., from small to large intestine), and among commensal microbes not facing any obvious selective pressures.

To date, characterizing the spatial structure of genetic diversity along the mammalian gut has been challenging because of the invasive nature of directly sampling the GI tract and given the limited bacterial biomass obtained from such attempts. However, these challenges can be circumvented using a combination of experimental and statistical approaches. First, the use of a humanized gnotobiotic mouse model for studying the gut microbiome enables the study of human gut-specific species of bacteria *in vivo* in a controlled manner^18^. Second, the use of mice overcomes the hurdle of invasive sampling in humans as the full gut contents can be extracted after sacrifice and then processed for sequencing. In addition, efforts to characterize genetic diversity along the gut are further supported by the ability to detect a range of microbes via shotgun sequencing that are otherwise undetectable using culture-based methods^19^. Finally, recent statistical innovations in phasing genotypes of individual strains from shotgun metagenomic data^20–22^ enables the ability to quantify strain frequencies and their evolutionary modifications.

In this study, we capitalize on these advances to assess the distribution of strains and evolutionary modifications along the gut. Specifically, we analyzed the frequencies of bacterial strains and the evolutionary modifications they carry along the gut of germ-free mice eight weeks after they were inoculated from a single healthy human stool sample (Figure 1B). We previously analyzed this same mouse dataset with 16S rRNA sequencing of luminal contents and found significant shifts in abundance of taxonomic family members along the gut^3^. By using metagenomic sequencing to analyze the same samples at a deeper level, we were able to estimate strain frequencies and identify evolutionary modifications. We report that the frequency of strains and evolutionary modifications for the majority of bacterial species was unexpectedly uniform across the gut. These results suggest that species abundance differences along the gut may play a greater role than genetic differences in responding to environmental gradients along the gut.

## Results

To understand the effect of gut region on microbial diversity, we orally gavaged eight germ-free Swiss Webster mice with a single stool sample (hereafter referred to as the inoculum) from a healthy human donor (Figure 1B). Luminal contents were collected from five intestinal regions in each mouse: the duodenum, jejunum, ileum, cecum, and colon. DNA was extracted and metagenomic sequencing was performed on each of the five mouse intestinal samples as well as the original human inoculum (Methods).

To measure diversity along the gut, we used a standard reference-based approach^23^ to quantify species abundances, SNV frequencies and gene copy number for the prevalent species present in these mice (see Methods for more details on the bioinformatic pipeline used). Summaries of species and genetic-level diversity have been previously quantified from stool with similar approaches (e.g., Garud et al., 2019)^20^, but here we investigate this diversity along the gut.

### Bacterial species diversity differs along the gut

**Figure 2:**
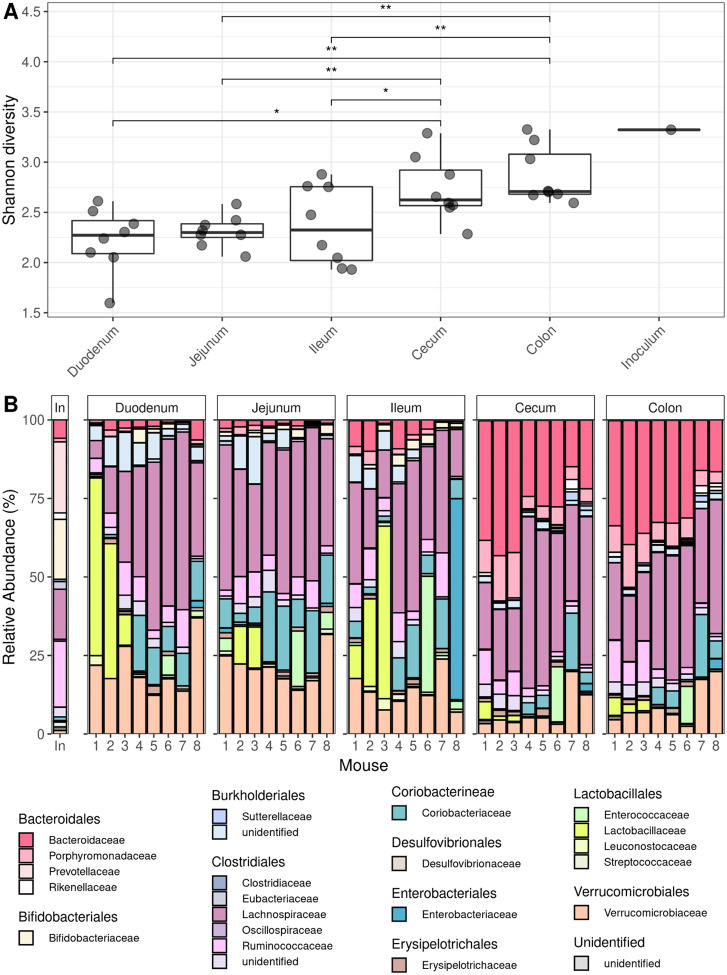
Taxonomic diversity increases and community membership changes along the length of the gut. **(A)** Alpha diversity (Shannon Index) estimates for bacteria in different regions of the gut. Two-sided Wilcoxon rank sum tests were conducted between gut regions (* indicates p ≤ 0.05; ** indicates p ≤ 0.01). **(B)** Relative abundance of bacterial families in the five gut regions and inoculum. Bacterial families are grouped by order in the legend.

First, we examined whether bacterial species diversity, measured using alpha diversity (the Shannon index), varied along the tract of the gut. We hypothesized that higher alpha diversity would be observed in the large intestine compared to other regions of the gut, as previous studies in humans^24^ and mice^18,25^ have found that species diversity is highest in the colon due to lower flow rates and the relatively high abundance of host- and food-derived complex polysaccharides^5,6,26^. As expected, gut region was a major driver of alpha diversity, with the large intestine displaying significantly higher diversity than all regions in the small intestine (average fold change per mouse = 1.22; Wilcoxon signed-rank test, p-value = 0.0156) (Figure 2A). We predicted that the alpha diversity of the inoculum would be greater than that of any gut segment, as species diversity would decline during inoculation due to not all human commensal species being able to colonize the mouse gut during the colonization process. Supporting our hypothesis, alpha diversity was lower in all gut samples (mean Shannon Index = 2.50) than in the inoculum (Shannon Index = 3.32), except for the colon sample of mouse 1 (Shannon Index = 3.41).

Next, we hypothesized that the relative abundance of specific bacterial members would differ along the gut in addition to diversity, as factors such as oxygen availability, antimicrobial peptides, and carbon-source favor different dominant taxa in the mammalian small and large intestines, respectively^1,2,27,28^. To quantify shifts in community composition along the gut, we coarsened our analysis by examining the relative abundance of bacterial families. We expected to see saccharolytic, obligate anaerobic taxa such as Bacteroidaceae, Ruminococcaceae, and Lachnospiraceae, dominate in the large intestine and rapidly dividing facultative anaerobes dominate in the small intestine^6,26,29^. Supporting our prediction, several families in the order Bacteroidales were enriched in the large intestine (Figure 2B), including Bacteroidaceae (average fold change per mouse = 15.2; Wilcoxon signed-rank test, p-value = 0.00781), Rikenellaceae (average fold change per mouse = 8.285; Wilcoxon signed-rank test, p-value = 0.0156), and Porphyromonadaceae (average fold change per mouse = 7.33; Wilcoxon signed-rank test, p-value = 0.00781). Bacteroidales is an order of anaerobic bacteria, in which many species are involved in breaking down complex sugars in the colon^30^. Meanwhile, we observed that facultative anaerobic families such as Lactobacillaceae^2^ had significantly higher abundance in the small intestine relative to the large intestine (average fold change per mouse = 9.22; Wilcoxon signed-rank test, p-value = 0.0225), as expected due to the relatively more higher oxygen environment of the small intestine enriching for facultative anaerobes relative to the oxygen-poor environment of the large intestine^26^.

Finally, confirming these differences in taxonomic composition between the small (duodenum, jejunum, and ileum) and large (cecum and colon) intestine, small and large intestinal samples formed discrete clusters (PERMANOVA, p-value = 0.01) in a principal coordinate analysis (PCoA) performed using beta diversity values (Bray-Curtis dissimilarity index) computed on species relative abundances (Supplementary figure 1). Together, these results are in line with previous work showing that the microbiota spatially segregates in taxonomic composition along the gut^3,26^.

### Bacterial genetic diversity along the gut

Having confirmed that taxonomic diversity varies spatially along the gut, we next investigated whether the same was true of within-species genetic diversity. To do so, we quantified nucleotide diversity (*π*) in the 30 most abundant species that were present in the inoculum and at least three mouse gut samples from at least two hosts (Methods) (Supplementary Figure 2). *π* is an estimate for the expected rate of nucleotide difference between any two genomes in a population. Across these species, nucleotide diversity measured from the inoculum was highly variable, ranging from 2.2 × 10^−4^ in *Odoribacter splanchnicus* to 1.2 × 10^−2^ in *Bacteroides vulgatus*, spanning two orders of magnitude. Diversity measured from the inoculum was highly correlated with diversity measured from the mouse gut averaged for each species (Spearman correlation, *ρ* = 0.85).

**Figure 3:**
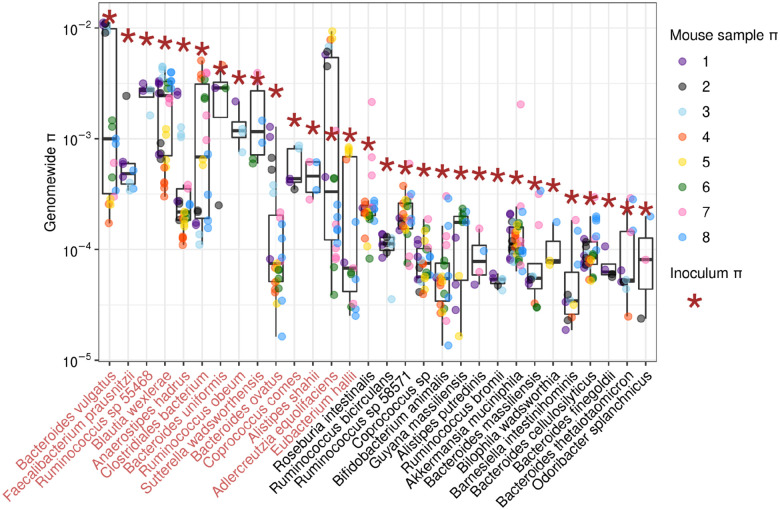
Nucleotide diversity measured in 30 gut commensal species. Each dot represents nucleotide diversity in a specific gut region within a specific mouse. Dots are colored based on which mouse they are from. Asterisks indicate nucleotide diversity measured in the inoculum. Species names in light red correspond to those species with *π* ≥ 1 × 10^−3^ in the inoculum.

To quantify variability in nucleotide diversity along the gut for a given species, we plotted the distribution of *π* measured across all gut segments for each mouse for each species (Figure 3). Variance in *π* within mice was substantially lower than variance between mice, especially for the species with the highest values of *π* in the inoculum. For example, for species with high inoculum *π* > 1 × 10^−3^, variance in *π* within mice was on average 4.53 × 10^−7^, while variance in *π* in the same gut region from different mice was on average an order of magnitude greater, 4.17 × 10^−6^. For these species with high inoculum *π*, average *π* values across mice were often bimodally distributed. For example, *π* values in the species *Bacteroides vulgatus* were either all clustered around 10^−2^ in some mice or 10^−3^ for other mice, but never spanned an order of magnitude within a single mouse (Figure 3). For the species with the lowest values of *π* in the inoculum (*π* < 1 × 10^−3^), variance in *π* along the gut was low not only within mice but also across mice (average within-host variance = 2.39 × 10^−8^; average between-host variance = 3.20 × 10^−8^).

We next asked what ecological or evolutionary mechanism promotes low variability in nucleotide diversity along the gut within mice versus high variability between mice, especially when diversity is high to begin with in the inoculum. High values of *π* (> 10^−3^) are generally inconsistent with the colonization and subsequent diversification of a single strain during a host’s lifetime, and instead support the presence of two or more strains that diverged long before colonization^20^. Low values of *π* are more supportive of the presence of a single strain. Therefore, the large range of *π* values observed across species in the inoculum indicate that some species are represented by multiple strains, and other species are likely represented by a single strain. Thus when *π* is high in the inoculum, it is likely that the gut of each individual mouse is consistently colonized by similar combinations of strains across different regions, while different mice are colonized by different combinations of strains, resulting in low variance in *π* within mice and high variance between mice. By contrast, when there is a single strain present in the inoculum, then there should be low variance in *π* within versus across strains because the same strain should be colonizing all mice and gut regions. In the next section, we further investigate the identities and frequencies of strains colonizing each mouse and gut segment for those species with high inoculum diversity levels.

### Strain frequencies are uniform along the gut within hosts

We asked whether the identities of strains colonizing different parts of the gut of a given mouse are the same or different when multiple strains are present in the inoculum, and moreover, whether their frequencies are variable along the gut. Variation along the gut would suggest that physical niche partitioning is important in maintaining multiple conspecific strains in the same host that might otherwise compete in the same physical niche, or that co-colonizing strains tend to be selected for different environmental pressures found along the gut.

To understand whether frequencies of co-colonizing strains of the same species vary along the gut, we inferred strain frequencies for the 14 species with levels of genetic diversity ≥ 1 × 10^−3^ in the inoculum, as these samples had sufficiently high enough diversity to potentially harbor at least two strains (Figure 3). To infer strain frequencies, we used an algorithm previously applied to metagenomic time series data^21,22^ (Methods). For the species *A. shahii*, *B. ovatus*, *C. comes*, and *F. prausnitzii*, only one strain was inferred to be present. For the rest of the species, we inferred that two strains were present (Figure 3; Supplementary Figure 3). We next asked whether the frequencies of strains for species with two strains was uniform or variable along the gut.

**Figure 4.**
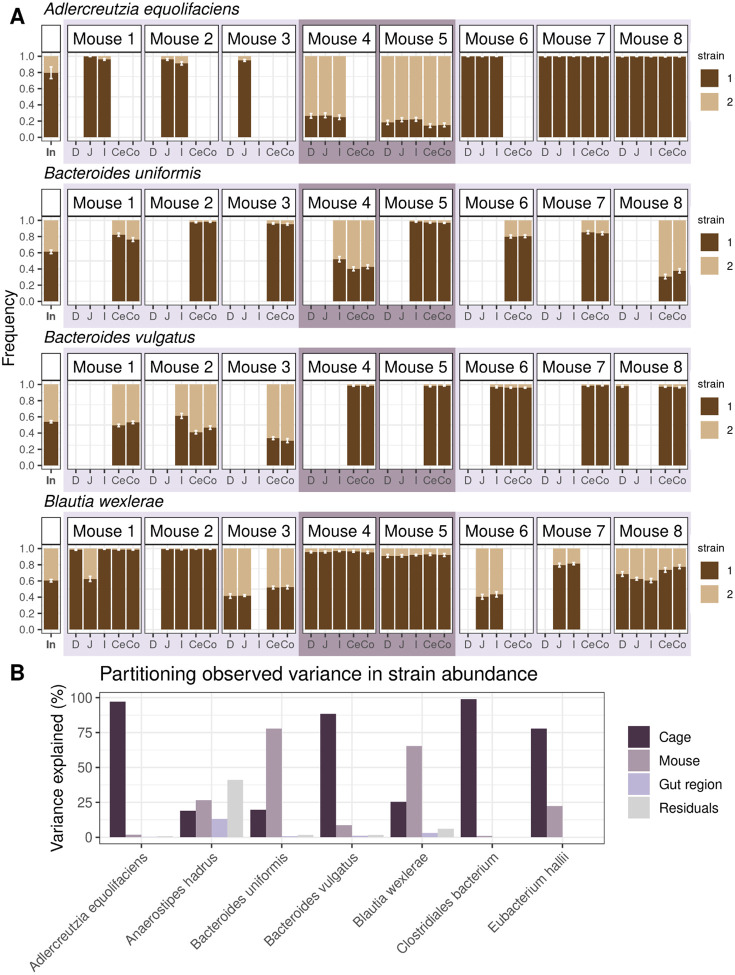
Similar strain frequencies are present along the length of the gut but may differ between mice housed in different cages. **(A)** Strain frequencies for four bacterial species detected in the guts of eight mice inoculated with the same human stool sample. A total of 11 bacterial species had high levels of inoculum nucleotide diversity (*π* ≥ 1 × 10^−3^), potentially indicating the presence of multiple strains. Using a previously developed strain-phasing algorithm^21,22^, we inferred the presence of two strains in the inoculum for seven of these species, and one strain for the remaining species (see Supplementary Figure 3 for the strain frequencies of the seven species not shown here). Error bars represent bootstrapped 95% confidence intervals. Mice 1–3 were co-housed in cage 1, mice 4 and 5 were co-housed in cage 2, and mice 6–8 were co-housed in cage 3, as indicated by the light and dark purple boxes. **(B)** ANOVA was used to quantify the amount of variance in major strain relative frequency explained by “cage”, “mouse”, and “gut region” in the seven species for which a sufficient number of high coverage samples were available to test the effect of all three variables on major strain abundance. Residuals of the ANOVA represent unexplained variance.

Application of the strain phasing algorithm revealed that frequencies of co-colonizing strains were relatively uniform across gut regions within hosts for most bacterial species and mice. For example, for the species *Blautia wexlerae*, the two strains were able to coexist at roughly 40:60% frequency along all five segments of the gut in mouse 8, and for the species *Adlercreutzia equilofaciens*, the two strains were able to coexist at roughly 20:80% frequency in all parts of the gut in mice 4 and 5 (Figure 4). We next quantified whether strain frequencies were more homogeneous within versus between hosts generally for all species in our dataset. To do so we performed a series of ANOVA tests to estimate the proportion of variance in major strain frequencies attributable to gut region (“gut region”), mouse identity (“mouse”), and cage identity (“cage”) (Figure 4B). We limited our analysis to the seven species detected in multiple cages, multiple mice per cage, and multiple gut regions (Methods) to assess the relative importance of all three variables: *A. equilofaciens*, *A. hadrus*, *B. uniformis*, *B. vulgatus*, *B. wexlerae*, *C. bacterium*, and *E. hallii*. Consistent with the observations of uniformity in genetic variation along the gut, gut region explained the lowest variance for all seven bacterial species (average variance explained = 2.70%), suggesting that gut region is less important in explaining strain frequencies than mouse identity or cage identity. By contrast, mouse and cage explained substantially greater variation (average combined variance explained = 90.0%). These observations support the hypothesis that strain frequencies along the gut are uniform and reveal that multiple strains are able to coexist in the same region of the gut. This implies some other ecological mechanism other than spatial segregation must be responsible for permitting strain coexistence within the same host without strains competing in the same physical niche.

One explanation for why strain frequencies are so similar along the gut may be that migration rates between gut sites are sufficiently rapid, such that strains quickly spread. Rapid migration rates may also facilitate between-host spread of strains, resulting in similar strain frequencies between mice in the same cage. Supporting this notion, strain frequencies were more similar among cohoused mice compared to mice housed in different cages, indicating that migration of strains between mice plays a role in homogenizing frequencies of strains. For example, for the species *A. equilofaciens*, which was present in multiple mice and cages, the within-cage variance of strain frequencies measured in the jejunum ranged from 1.65 × 10^−6^ in cage 3 to 1.29 × 10^−3^ in cage 2, whereas the between-cage variance was at least two orders of magnitude greater, 1.18 × 10^−1^ (Figure 4). These results align with previous studies that have found coprophagic behavior in mice can facilitate the spread of strains and adaptive variants in *Bacteroides thetaiotaomicron*^31^ and *E. coli*^14,32^ between co-housed mice, enforcing similar strain frequencies between mice^14^. However, not all species have similar within-cage strain frequencies (e.g., *B. uniformis*), suggesting barriers to migration in these cases. This species-specific migration effect is reflected in the fact that “cage” explains most of the variance in strain frequency for four species, but very little variance in the remaining three species (Figure 4B).

Together, these results indicate that co-colonizing strains do not need to occupy physically distinct niches to colonize the same host. Moreover, strain frequencies are relatively uniform along the gut. This uniformity may be driven by rapid migration rates, which also likely results in greater similarity in strain frequencies among mice of the same cage versus different cages due to social transmission.

### Evolutionary changes spread throughout the gut

Even if bacterial strain frequencies are relatively homogenous along the gut, it is possible that individual genetic variants within species are locally restricted along the gut, potentially due to local adaptation. To assess the possibility of local adaptation, we first identified extreme SNV frequency changes between pairs of samples and examined whether they are spatially segregated along the gut or if they too are homogeneously distributed.

SNV frequency changes between gut regions can arise within a host as a consequence of (1) evolutionary changes (e.g., adaptation), (2) fluctuations in frequencies of genetically distinct strains, or (3) sampling error. Here, we are interested in SNV frequency changes that arise as a consequence of evolutionary changes and not strain fluctuations or sampling error. To distinguish SNV frequency changes arising due to evolution as opposed to strain fluctuations, we can infer the genotype of one of the strains present in the sample, or ‘quasi-phase’ the genotype^20^ and then track SNV frequency changes on the background of that genotype between pairs of samples. Doing so can allow us to detect new mutations and recombination events modifying a resident lineage as opposed to alleles rising to high frequency due to strain fluctuations. To distinguish SNV frequency changes due to evolution (e.g., adaptation) versus sampling error, we can consider only large allele frequency changes (*f* ≤ 0.2 in one sample to *f* ≥ 0.8 in another sample) at sites with a minimum read coverage of 20 or more in both samples, which are unlikely to arise due to sampling error or drift^20,33^. Previously, we showed that the genotype of the dominant lineage in samples with a single, high-frequency strain can be confidently quasi phased^20^, and that evolutionary changes with large allele frequency changes can subsequently be detected.

**Figure 5:**
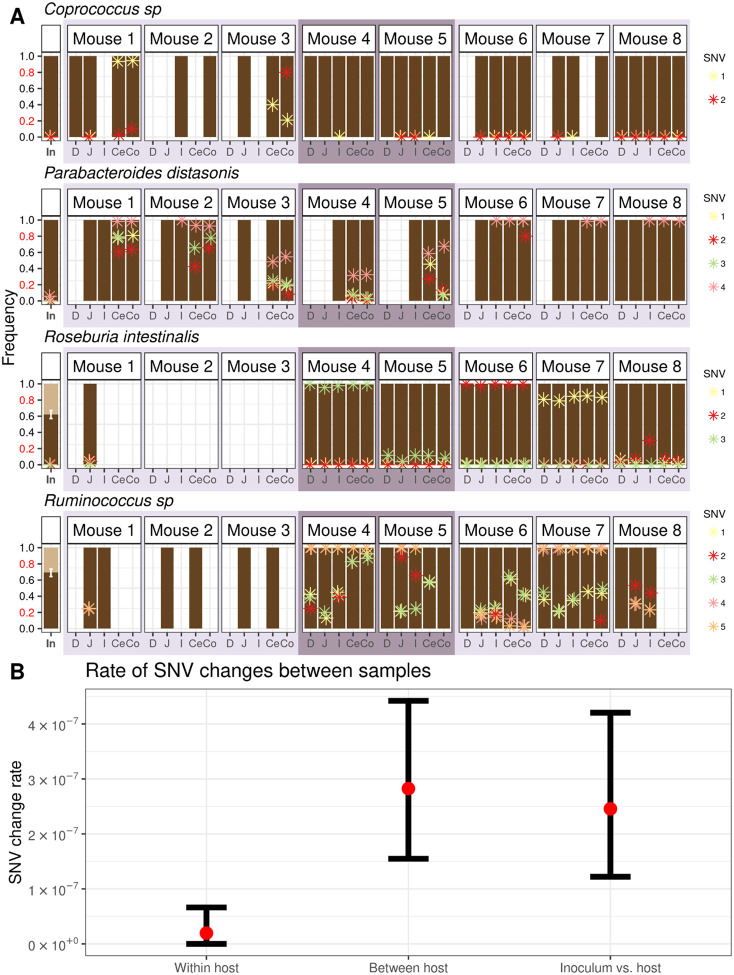
Evolutionary changes are uniformly found along the gut. **(A)** SNVs exhibiting extreme allele frequency changes (allele frequency *f* ≤ 0.2 in one sample to *f* ≥ 0.8 in another sample) between any pair of samples sharing the same QP strain are shown for *Coprococcus* sp., *P. distasonis*, *R. intestinalis*, and *Ruminococcus* sp. Asterisks represent the frequency of a given SNV, with each SNV within a species having its own unique color. Samples lack asterisks for a particular SNV when those loci do not meet the minimum coverage requirement of 20 reads to infer frequency. When two strains of the same species are present, strain abundances of co-colonizing strains are represented by two different shades of brown (see Figure 4 for legend). The remaining 12 species in which SNV frequency differences were detected are shown in Supplementary Figure 4. **(B)** Comparison of the rate of SNV frequency changes within mice, between mice, and between inoculum and mice. A bootstrap method was used to compare the rate of SNV frequency changes. Colored in red are the observed SNV frequency change rates. Bars represent bootstrapped 95% CIs.

Using this method, we identified 366 quasi-phaseable (QP) genomes from 62 bacterial species present in the intestinal contents of the eight mice and the inoculum (Supplementary Figure 1). Next, we detected SNV frequency changes between pairs of QP genomes. To avoid comparing distantly diverged lineages that were not descended from the same inoculum strain, we only considered pairs with <50 SNV frequency changes. These pairs were between the inoculum and any mouse (10 bacterial species), different gut regions *within* mice (spanning 33 bacterial species), and gut regions *between* mice (28 bacterial species). We observed a total of 65 unique SNV frequency changes across all possible pairs of QP genomes in our dataset (Figure 5A, Supplementary Figure 3, Supplementary Table 3).

There were 11 SNV frequency changes observed to arise in mice relative to the inoculum, potentially indicative of adaptation during the host colonization process. Only one of these SNVs showed spatial differentiation, indicating that advantageous SNVs tend to spread along the gut. Since not all SNVs observed within mice had sufficient coverage to be analyzed in the inoculum, we next examined SNV frequency changes arising between gut segments. Supporting the observation that SNVs spread along the gut, we observed only 4 unique *within-host* SNV frequency changes in 3 out of the 33 species. By contrast, we observed 57 unique *between-host* SNV frequency changes in 15 of the 28 species. The rate of SNV frequency changes observed between hosts was significantly higher than that within hosts (bootstrapped 95% confidence interval for between-host SNV changes is 1.54 × 10^−7^ differences/bp - 4.42 × 10^−7^ differences/bp; bootstrapped 95% confidence interval for within-host SNV changes is 0 differences/bp - 6.62 × 10^−8^ differences/bp) (Figure 5B). The low rate of SNV frequency changes within hosts suggests that advantageous SNVs overwhelmingly transmit along the gut within hosts.

To understand whether SNVs spreading along the gut showed additional signals of being adaptive beyond experiencing extreme allele frequency changes on short time scales, we asked if the same SNV changes arise in multiple mice, as parallelism is a hallmark of adaptation^34–38^. Adaptive variation can arise in multiple mice via independent recurrent *de novo* mutation or the parallel rise in frequency of standing genetic variation (SGV) already present in the inoculum^38^. Of the 21 unique SNVs that were observed to undergo an extreme allele frequency change between any pair of samples and had sufficient coverage in the inoculum (minimum of 20 reads), 15 had non-zero frequency in the inoculum, indicating that SGV is an important source of genetic variation in these mice. Of these, five nonsynonymous and one synonymous SNVs were observed to undergo parallel sweeps from low frequency in the inoculum to high frequency in multiple independent cages. One of these parallel SNVs was in *P. distasonis* in a gene encoding a TonB-dependent transporter and rose from low frequency in the inoculum (*f* = 0.057) to high frequency in multiple mice in all three cages (average *f* = 0.319 in mouse 4 to average *f* = 1.00 in mouse 6), consistent with parallelism. TonB-dependent transporters are ubiquitous outer membrane-associated proteins involved in nutrient transport^39^, and a common target of adaptation in the human gut microbiome^37,40,41^. Notably, all the SNVs observed to undergo parallelism were not found to be differentiated along the gut within hosts. This fact would seem to suggest that even when SNVs are adaptive, they are either broadly adaptive to the entire gut, or alternatively have high migration rates that outweighs local selection pressures, perhaps due to mixing and peristalsis along the gut^42^.

## Discussion

In this study, we set out to characterize how genetic diversity varies along the gut. To do so, we analyzed the frequencies of bacterial strains and evolutionary changes along the guts of eight mice inoculated with the same human-stool-derived inoculum. In sharp contrast to the species-level heterogeneity reported here and in previous studies^3,6,26,27,43–51^, we found substantial genetic homogeneity along the gut (Figure 4, Figure 5, Supplementary Figure 3, Supplementary Figure 4). These results indicate that spatial segregation of strains is not a requisite for their coexistence and that environmental gradients do not necessarily generate large strain fluctuations or allele frequency differences along the gut lumen.

Spatial uniformity was generic to all of the 35 species analyzed in our study, indicating this was not a phenomenon driven by only a few species (Figure 4, Figure 5, Supplementary Figure 3, Supplementary Figure 4). Uniformity likely arose due to rapid migration rates of bacteria between gut regions and, in several instances, also between mice (Figure 4A–B, Supplementary Figure 3, Supplementary Figure 4). This latter conclusion is supported by the observation that strain frequencies are more similar between co-housed mice versus mice housed in different cages, consistent with recent work showing extensive social transmission of bacteria among co-housed mice^14,32^. While it is possible that the same evolutionary changes may be arising in different locations along the gut independently of each other, SNVs are usually found at similar frequencies along the gut suggesting instead migration of variants arising from a single origin. These results are consistent with recent work showing that fluid flow along the gut is unlikely to generate stochastic allele frequency changes in the gut microbiome^33^ and that there is significant mixing of contents along the gut^42^.

Though our study is the first to document uniform strain frequencies along the length of the mouse gut, the stable coexistence of strains at similar frequencies over time has been documented previously in human fecal microbiomes^22,24^ and in experimental evolution studies^52,53^. Stable coexistence of conspecific strains is thought to be facilitated at least in part by metabolic niche partitioning^54^. Our findings support this by ruling out the need for strains to be spatially segregated to reduce competition.

The 65 SNV frequency differences we detect are likely adaptive due to their large allele frequency changes accruing on short time scales^20^, their parallel rise to high frequency in multiple cages for a subset of these, and the enriched nonsynonymous to synonymous ratio for parallel sweeping SNVs. The spread of adaptive alleles along the gut thus implies that they may be broadly adaptive rather than being locally adaptive. Alternatively, adaptive alleles that arise due to local selective pressures may be spreading widely due to rapid migration rates even if only advantageous in specific gut regions. In our study, we did not focus on smaller allele frequency changes as these could be more easily confounded due to sampling error with shotgun data, and are less likely to have arisen due to adaptation. However, in future work, understanding how finer-scale neutral genetic variation is distributed along the gut may reveal different spatial patterns than the ones we observed for extreme frequency SNV changes. Indeed, in two recent human studies analyzing metagenome assembled genomes, potentially neutral evolutionary changes in a limited number of species were found to appear in local regions along the gut^16,17^, and so it remains to be seen how common this phenomenon is.

Importantly, our study has several limitations that require further investigation to ensure the generalization of our findings. First, in our system, we investigated a single human microbiota inoculated in mice under a highly controlled experimental setting. While this controlled design minimizes variability, it may not fully reflect the complexity of microbial dynamics in more natural or diverse environments. Furthermore, because the inoculum microbiota originated from a human sample, these strains may have impaired interactions with the mouse host, potentially precluding spatial organization at the SNV level. Although we observed significant spatial differences at the species level, suggesting these effects may be minor, future studies using conventional mouse microbiota would help determine whether host adaptation plays a critical role in spatial organization at the subspecific level.

Additionally, some spatial organization may be averaged out due to coprophagy resulting in microbial transfer between individuals or autocoprophagy resulting in microbial transfer from the lower GI to the upper GI tract of the same individual. However, we think these effects are unlikely to explain the uniformity we observed, given the robust species-level spatial organization.

This study focuses on healthy mice, but localized inflammation or environmental changes could further alter microbiota spatial organization. In future work, it will be interesting to investigate if spatial structure at the genetic level arises in diseased hosts. Intestinal disorders such as inflammatory bowel disease (IBD) are known to generate local inflammation phenotypes and disrupt gut microbiome composition^55,56^ in specific gut segments^4,57,58^. Additionally, in a recent mouse study, adaptive SNVs in a species of pathogenic bacteria were found to appear locally in different colonic tissues, enabling translocation of a pathogenic bacteria from the colonic lumen to the mucosa, and subsequently to the liver^9^. Thus, in pathogenic bacteria and disease scenarios, we might observe specific strains or local adaptations associated with these acute conditions along the gut, supporting our original hypothesis that environmental heterogeneity might induce genetic diversity along the gut.

Finally, in future work, applying a finer-resolution sampling strategy, either in time or space, may reveal bacterial genetic heterogeneity that was not observed in this study. For example, sampling the gut prior to eight weeks post-inoculation might have captured the spread of alleles before they migrated to all gut regions. Additionally, while this study focused on the gut lumen at five sites along the gut and found genetic variation to be uniformly distributed, other physiological structures found in the mammalian gut might give rise to more heterogeneous spatial distributions. For example, different crypts in the intestinal epithelium could potentially be occupied by distinct strains, similar to that of skin pores^59^. Crypts are known to have communities distinct from the lumen^43,60^, and may perform ecological filtering at the strain and nucleotide-level as well. Similarly, the mucosa displays different biogeography from the lumen^44^, and mucosal surfaces are known to generate population structure in culture^61^, promote site-specific binding in certain bacterial species that have co-evolved with their hosts^62^, and drive adaptive differentiation relative to populations in the gut lumen^9^. However, the technical challenges in obtaining sufficient bacterial biomass from crypts or mucosa is significant^63^. As innovations develop in sampling and host-depletion strategies (e.g., Wu-Woods et al., 2023)^17^, heterogeneity will be able to be investigated at finer scales.

In conclusion, our findings demonstrate that bacterial strains and SNVs are uniformly distributed along the gut lumen of germ-free mice inoculated with a human stool sample, in contrast to the bacterial heterogeneity observed at higher taxonomic levels. These findings indicate that species-level diversity may in fact play a greater role in responding to environmental heterogeneity along the gut than genetic diversity. In the future, understanding the spatial dynamics of strains and evolutionary changes in diverse cohorts including diseased individuals, as well as at finer temporal and spatial scales, may shed light on their functional importance along the gut.

## Methods

### Experimental design

To understand the effect of spatial location on microbial diversity along the gut, we performed shotgun sequencing on the luminal contents of mice we previously analyzed with 16S rRNA sequencing^3^. All animal experiments were conducted in accordance with the ethical guidelines of the University of British Columbia’s (UBC) animal care procedures, following protocol numbers A19–0078 and A19–0122 approved by the Animal Care Committee. Swiss Webster male and female mice at nice weeks of age were used and were provided with an autoclaved standard diet (Purina LabDiet 5K67). Briefly, eight germ-free Swiss Webster mice were orally gavaged with the same human stool sample from a healthy adult human previously labeled TL1^3^. The eight mice were housed in three different cages in the following configurations: cage 1 had mice 1–3 (sex male), cage 2 had mice 4 and 5 (sex male), and cage 3 had mice 6–8 (sex female). Mice were equilibrated for six weeks on a standard rodent diet (LabDiet 5k67). Subsequently, mice in cages 2 and 3 were exposed to a fiber-rich diet of 30% guar gum (TestDiet 5BSE) for two weeks while the rest continued on the standard diet. With this experimental set up, we were able to ascertain replicability and generalizability of the genetic homogeneity observed along the gut in three independent cages with varying diets and sexes, as well as contrast within-host dynamics with across-host dynamics.

Mice were sacrificed two weeks post equilibration with the diet using carbon dioxide with secondary cervical dislocation, and the luminal contents were collected from five intestinal regions: the duodenum, jejunum, ileum, cecum, and colon. The intestines were carefully sectioned using a razor blade into the corresponding regions and the contents were squeezed into 1.5 mL-microcentrifuge tubes. DNA extraction was performed on luminal content samples and the original inoculum (TL1) from 96-well plates using the DNeasy PowerSoil Pro Kit (Catalog number: 47016). For more information on the approach used, see the methods section of Ng et al., 2023^3^.

### Metagenomic Sequencing

Metagenomic sequencing was performed on luminal content samples and the inoculum using a NovaSeq with 150 bp paired end reads. Mouse samples were sequenced to a depth ranging from 23M-136M reads (median = 33M reads), and the inoculum was sequenced to a depth of 119M reads (Table S1). Samples from mice 7, 8, and the inoculum were sequenced in a separate batch than from samples from mice 1–6 at the highest end of the range of sequencing depth.

### Metagenomics pipeline

Metagenomic Intra-species Diversity Analysis System (MIDAS; version 1.2, downloaded November 24, 2021)^23^ was used to calculate relative species abundances, gene copy number variants (CNVs), and single nucleotide variants (SNVs). While the output of MIDAS was generated with permissive parameters, as described below, we subsequently applied several stringent post-processing filters to rule out metagenomic artifacts and to accurately estimate species, CNVs, and SNVs. The post processing pipeline we use is briefly described below and reflects our pipeline developed in Garud et al., 2019^20^.

### Estimating species relative abundance

Briefly, species presence and abundance was estimated by mapping reads to 15 single- copy marker genes from 5,952 representative species^23,64^. Reads were mapped with default MIDAS settings. Species with an average single copy marker gene coverage ≥3 reads were marked as present within a sample. These read coverages were then used by MIDAS to compute species relative abundance.

Next to identify CNVs and SNVs, we defined a mouse-specific reference database of species to map reads to. Like in Garud et al. 2019^20^, our goal in defining this database was to have as many genomes necessary to map reads accurately while excluding an excess of genomes not actually present in our sample. In particular, we wanted to prevent read *donating*, which could occur when a species that is truly present but not represented in the database, or read *stealing*, which could occur when a species that is not truly present but attracts reads erroneously. As such, we defined a private database for each mouse to include any species marked as present in any gut segment.

### Quantifying gene copy number

To minimize read mapping errors, we identified genes with abnormally high gene copy number values, which may be potentially indicative of erroneous read recruitment. MIDAS estimates CNVs by mapping reads to a *pangenome* consisting of several sequenced isolates belonging to the same species. Average gene coverage was calculated as the number of reads mapping to that gene normalized by gene length. MIDAS computes copy number, *c*, as the ratio between a gene’s average coverage and the median coverage of a set of 15 single-copy marker genes. Genes with a copy number *c* > 3 in at least one sample in our study were excluded as they were potential candidates for stealing. To exclude any additional genes that may suffer from erroneous read recruitment, we further filtered out any genes with a copy number *c* > 3 in the human samples analyzed in Garud et al., 2019^20^. Finally, any genes sharing ≥ 95% average nucleotide identity with any other gene across species boundaries as identified in a set of “blacklisted” genes in Garud et al., 2019 (section A.iv)^20^ were excluded to further prevent erroneous read mapping.

### Quantifying SNV frequencies

Finally, we estimated SNV frequencies for each species in each sample. Downstream, these frequencies were used to estimate nucleotide diversity, identify QP samples, and quantify SNV frequency differences between pairs of QP samples. To estimate SNV frequencies, reads were mapped to representative reference genomes using default MIDAS mapping thresholds and arguments^20^.

To obtain accurate estimates of SNV frequencies, we imposed coverage requirements. MIDAS reports the read coverage *D* for each site in the reference genome for each sample. We excluded any sample with a mean *D* < 5X across all protein coding sites, as was done in Garud, Good et al. 2019. We further excluded from analysis species non-zero coverage at ≥ 40% of reference sites within a sample. Additionally, to minimize the possibility of any mapping errors, sites with a coverage value < 0.3X mean *D* or > 3X mean *D* were excluded, as was done in Garud et al., 2019^20^. Finally, SNV frequency differences were identified only if successive values of coverage between two samples from the same mouse were within a factor of three, as we expect that coverage should be relatively constant within a mouse. Below, additional coverage filters were imposed for various calculations.

SNVs were annotated as synonymous and nonsynonymous based on the reading frames of genes as annotated in the PATRIC database^65^.

### Calculating alpha and beta diversity of gut samples

Alpha diversity was computed using the Shannon diversity index as implemented by the Vegan package in R^66^. To assess if Shannon diversity between gut regions was significantly different, we used a paired Wilcoxon signed-rank test comparing pairs of samples from one region versus the other from the same mouse. To calculate fold change in alpha diversity between the small and large intestines, alpha diversity was averaged across small intestinal segments (duodenum, jejunum, and ileum) and large intestinal segments (cecum and colon), and the per mouse fold change was calculated as the ratio of the averaged large intestinal and small intestinal alpha diversity values. The fold change values presented in the main text represent the average of these per-mouse estimates.

To produce the stacked bar plots in Figure 2B, species abundances within taxonomic families were summed. Species with less than 0.1% abundance were excluded. To test for differential abundance of specific families between the small and large intestine, relative abundances for each family were averaged across the small intestine (duodenum, jejunum, and ileum) and large intestine (cecum and colon) in each host and a paired Wilcoxon signed-rank test was applied in which samples from the same mouse were paired. Fold change in relative abundance between the large and small intestine is calculated in the same manner as with alpha diversity, using relative abundance instead of alpha diversity.

To perform the PCoA analysis in Supplementary Figure 1, relative species abundances were used to calculate the Bray-Curtis dissimilarity index (i.e., beta diversity) between all pairs of samples using the Vegan package in R. Bray-Curtis dissimilarity indices were used as the proximity matrix in PCoA, and samples were visualized on the first two dimensions. Bray-Curtis dissimilarity indices were also used to perform a permutational multivariate analysis of variance (PERMANOVA statistical test; Vegan package) to determine whether species composition was significantly different between the small and large intestine.

### Calculating nucleotide diversity

For every sample, we estimated *π*, a population-level metric of nucleotide diversity that represents the probability of choosing two different alleles at a randomly chosen base pair in the genome. To do so, we used the formula for nucleotide diversity applied in Schloissnig et al., 2013^67^, which accounts for total read counts:

$$\pi (S,G)=\frac{1}{\left|G\right|}\overset{\left|G\right|}{\underset{i=1}{\sum }}\underset{B_{1}\in \left\{\text{ATGC}\right\}}{\sum }\underset{B_{2}\in \left\{\text{ATGC}\right\}\setminus B_{1}}{\sum }\frac{x_{i,B_{1}}}{D_{i}}\frac{x_{i,B_{2}}}{D_{i}-1}$$

Here, *S* is the sample; *G* is the genome of the focal species, with |*G*| being its size; *i* is the locus in the genome; *B*_1_ and *B*_2_ are the reference and alternative alleles, respectively; $x_{i,B_{j}}$ is the number of reads with allele *B*_*j*_ at locus *i*; and *D*_*i*_ is the total read depth at locus *i*. This formula is an extension of a previously proposed *π* estimator on next generation sequencing data devised by Begun et al., 2007^68^. Sites with read coverage less than four were excluded from *π* calculations, such that |*G*| is equivalent to the total number of sites with adequate coverage rather than the total size of the genome.

Samples from mice 7 and 8 were sequenced to a substantially higher depth compared to samples from mice 1 through 6 (Supplementary Table 1), resulting in elevated levels of coverage per bp for bacterial species in mice 7 and 8. As a consequence, there was an increased opportunity to detect higher levels of nucleotide diversity in mice 7 and 8 merely due to differences in coverage. To be able to compare nucleotide diversity levels between mice, we applied a random down sampling per nucleotide site such that the depth of mice 7 and 8 matched the median coverage of mice 1 through 6.

Distributions of *π* in Figure 2 were plotted for the 30 species which met the following criteria: minimum coverage of four reads in the inoculum and at least three mouse samples representing at least two different mouse hosts. Species were only considered in samples in which at least 500,000 nucleotides met the aforementioned minimum coverage threshold.

### Inferring strain frequencies

Previous work has shown that hosts tend to be colonized by multiple genetically distinct strains (typically 1–4) of the same species^20,69^. When levels of recombination between strains within a host is sufficiently low, these strains may co-exist as genetically distinct subpopulations^21^. Typically, two randomly drawn strains from different hosts harbor on order 10^3^ – 10^4^ SNV changes^20,37,67,70^ and two strains residing within a host are expected to be similarly diverged^20^. We leverage this expected divergence between strains to distinguish between the strain genotypes and infer their frequencies.

Inferring strain genotypes and frequencies from short read sequencing data is a difficult problem because linkage between co-segregating SNVs residing on the same strain backbone are destroyed by the fact that they are unlikely to reside on the same short read. Even perfectly linked SNVs may be difficult to assign to the correct strain given the potential for sampling noise. However, recent work has shown that strain inference is feasible when there are many samples collected from the same hosts, as alleles belonging to the same strain will display highly correlated allele frequencies across samples^21,22,53,71–73^.

Here we leverage recent work by Roodgar et al., 2021^21^ and extended by Wolff et al., 2023^22^ to infer strain identity and frequency. These papers inferred strain genotypes and frequencies from *temporal* data. Here, we use *spatial* data both within mice and across mice to infer strain genotypes and frequencies. Since all mice were inoculated with the same human sample, we treat each sample in our dataset as one would temporal samples collected from the same host

Briefly, the approach clusters SNVs segregating on the same strain background by detecting correlations between SNVs with highly similar allele frequencies across samples. The degree of correlation between a pair of SNVs *i* and *j* is measured by distance metric *d* between allele frequency trajectories ${\hat{f}}_{i}$ and ${\hat{f}}_{j}$:

$$d\left({\hat{f}}_{i},{\hat{f}}_{j}\right)=\frac{1}{S}\sum_{S=1}^{S}\frac{2\left(D_{is}+D_{js}\right){\left({\hat{f}}_{is}-{\hat{f}}_{js}\right)}^{2}}{\left({\hat{f}}_{is}+{\hat{f}}_{js}\right)\left(1-{\hat{f}}_{is}+1-{\hat{f}}_{js}\right)},$$

where *S* is the number of samples, ${\hat{f}}_{is}$ and ${\hat{f}}_{js}$ are the frequency of the alleles in a sample *s*, and *D*_*is*_ and *D*_*js*_ represent the read depth of the alleles in sample *s*. SNVs that are in perfect linkage with one another are expected to have a *d* = 0.

To ensure that high-quality and informative sites were included in our stain inference procedure, we only considered loci that had a read coverage of *D* ≥ 10 and were polymorphic in at least 20% of samples in which the species was detected. Filtering out non-polymorphic loci prevented the algorithm from generating clusters at allele frequencies of 1 or 0 that reflect low frequency variation within a strain population as opposed to actual genetically distinct strains. In addition to the filters used by Wolff et al., 2023, we required minor alleles to have a read support of at least four reads to prevent rare mutations from erroneously giving rise to low-frequency clusters.

Detecting correlated clusters of SNVs belonging to the same strain background can be confounded without knowing if the alternative or reference allele belongs to one strain versus another. To address this issue, as done in Roodgar et al. 2021^21^, we set *d* between a pair of loci to the minimum of $d\left({\hat{f}}_{i},{\hat{f}}_{j}\right)$ and $d\left({\hat{f}}_{i},1-{\hat{f}}_{j}\right)$ to ensure that polarization differences (i.e., calling an allele frequency 0.2 instead of 0.8, based on the reference allele used in the reference genome) between loci did not prevent them from being clustered together.

To identify SNVs linked on the same strain background, we applied a greedy, network-based algorithm to extract large cluster SNVs that are highly correlated allele frequencies across samples. The algorithm begins by forming a network in which nodes represent SNVs, with pairs SNVs connected by an edge if they have a distance *d* < 3.5, which represents a maximum distance threshold below which pairs of SNVs are likely to be linked on the same strain background, as demonstrated in Roodgar et al., 2021^21^. Next, the algorithm identifies a focal SNV as the SNV with the maximum number of connections (i.e., edges), and extracts all SNVs connected to it. This cluster of SNVs was designated as a strain if it included at least 10^3^ SNVs, as this represents the lower bound for the typical number of alleles that are expected to segregate between genetically distinct strains^20,37,67,70^. Note that SNVs connected to the focal SNV were only extracted if they were also connected to at least 25% of other SNVs within a cluster. SNVs included in the initial cluster were removed from the overall set of variable sites, and the clustering process was repeated until no additional clusters of at least 10^3^ SNVs were identified. Strain frequencies were then inferred as the mean SNV allele frequencies in each cluster (Supplementary Figure 4). However, despite requiring strain clusters to have a minimum of 10^3^ SNVs, not all SNVs met our coverage requirement of *D* ≥ 10 across all samples. Therefore, we only report strain frequencies for samples in which every cluster has at least 100 high coverage SNVs (D ≥10) to ensure that the clusters were well-supported.

In a scenario when only one strain is present, we expect *zero* clusters to be detected. In a scenario in which two strains are present, we expect only *one* cluster, representing the alleles segregating between the two strains. The relative frequencies of the two strains can be inferred as $\bar{f}$ and $1-\bar{f}$, where $\bar{f}$ is the mean frequency of the clustered SNVs. In a scenario in which three strains are present, we expect *three* clusters, representing the SNV segregating between each of the three pairs of strains. This third scenario did not occur in our dataset.

We visually inspected all inferred clusters. In one case, *B. uniformis*, the strain inference algorithm inferred *two* clusters (Supplementary Figure 5A). However, the output of two clusters is inconsistent with a scenario of either 1, 2 or 3 strains colonizing and instead may be arising from high variance in allele frequency. It was visually apparent in this case that the two clusters likely represented the same group of SNVs segregating between two strains’ genetic backgrounds because of their highly correlated trajectories, but were separated into two distinct clusters due to there being a large variance in individual SNV frequency. In this singular instance, we reclustered by using the mean SNV frequency across all samples as a focal SNV (Supplementary Figure 5B). This method produced a single cluster. The algorithm was able to infer strain frequencies for all other species without the need for *ad hoc* modifications to the pipeline.

We generated 95% confidence intervals for strain frequencies in each sample using a stationary bootstrapping approach^74^. Specifically, 1,000 bootstrap samples each consisting of 100 SNVs were drawn with replacement from each strain cluster within each metagenomic sample, and subsequently the mean of the subsampled SNV frequencies in each bootstrap sample was computed. The upper and lower bounds of the 95% confidence interval was calculated as the 2.5 and 97.5 percentile of the bootstrapped strain frequency distribution.

In some instances, only a single strain was inferred for a bacterial species across all samples, despite the inoculum displaying high nucleotide diversity for that species consistent with multiple strains being present. This phenomenon may occur when there are multiple strains in the original inoculum, but one of those strains successfully colonizes the mice. As a result, the algorithm would be unable to detect correlations between the SNVs unique to the non-colonizing strains across many samples, and therefore fail to detect those strains. We identified when this might be happening by looking for species in which only a single strain was inferred, but which had at least 1,000 SNVs in the inoculum that were polymorphic and had a read support of four reads for the minor allele. When this scenario occurred, we assumed two strains were present in the inoculum and inferred the strain frequency *f*_*strain* 1*,inoculum*_ of the first strain as the mean of the allele frequencies of these polymorphic SNVs in the inoculum, and the strain frequency *f*_*strain* 2*,inoculum*_ of the second strain as 1 − *f*_*strain* 1*,inoculum*_. We generated 95% confidence intervals by applying the aforementioned bootstrapping approach to the polymorphic SNVs identified in the inoculum.

### Performing ANOVA of major strain frequencies

To quantify the variance in major strain frequency across samples, we applied an ANOVA approach. For the species in which co-colonization occurred in mice, the major strain was designated as the strain at frequency *f* > 0.5 in the inoculum. The centered log ratio (CLR) transformation was applied to major strain frequency in all samples in which that species was detected, a commonly used approach to remove compositional artifacts when applying non-compositional approaches such as ANOVA^75,76^. For each species, we built an ANOVA model using the Stats package in R. CLR-transformed major strain frequency was the dependent variable, while cage, mouse, and gut region were independent variables. Variance explained was calculated as the sum of squares between groups corresponding to each variable divided by the sum of squares total. To ensure that the amount of variance explained by each dependent variable was properly quantified, we ensured that each species was detected across at least two cages (with the species detectable in at least two mice in those cages), at least two mice (with the species detectable in at least two gut regions in those mice), and two gut regions (with the species detectable in these same gut regions in at least two mice).

### Identifying quasi-phaseable lineages

SNV frequency differences between samples can arise due to evolution, or due to shifts in the frequency of co-colonizing strains, which are likely to harbor thousands of differing nucleotides between them. To distinguish between SNV frequency differences that arise due to evolution versus those arising from strain frequency shifts, we identified quasi-phaseable (QP) lineages using an approach developed in Garud et al., 2019^20^. By inferring QP lineages, we then were able to confidently infer evolutionary changes.

Briefly, the approach works by identifying samples in which a particular species has a single dominant strain at *f* ≥ 0.8, hereafter referred to as a QP strain. In such samples, the allele corresponding to the dominant strain can be inferred with statistical confidence. Specifically, the alleles with frequency *f* ≥ 0.8 at genomic loci with coverage *D* ≥ 20 are assigned to the QP strain. Only samples with a median coverage of 20X are processed through the QP pipeline. Employing such stringent coverage and allele frequency thresholds ensures that the incorrect allele is not inferred, as it is statistically improbable that sampling error would give rise to the incorrect allele^20^.

One important alteration was made to application of the QP pipeline in this study relative to its application in Garud et al., 2019^20^. Normally, the QP pipeline designates a sample as having a QP strain if the number of intermediate frequency SNVs (i.e., SNVs with frequency 0.2 < *f* < 0.8) which a species harbors within the sample are 10% or less than the number of SNV differences that are typically observed for that species between independent hosts. This heuristic arises from the assumption that independent hosts should harbor genetically distinct strains that have diverged over long evolutionary timescales, and is born out by empirical evidence^20^. The 10% threshold is calibrated independently for each species, as genetically distinct strains of one species are not necessarily expected to show the same level of nucleotide differentiation as genetically distinct strains of a different species. In previous applications of the QP pipeline, the number of SNV differences between independent hosts has been assessed from the dataset actually used in those studies. For example, Garud et al., 2019^20^ studied evolutionary dynamics within 693 unique individuals sampled as a part of the human microbiome project^77,78^, the TwinsUK registry^79^, a cohort of chinese subjects^80^, and four additional young twins^81^. Because the vast majority of hosts included in this compiled dataset were unrelated (and in some cases, on different continents), the assumption that average rate of between-host SNV differences represented the amount of differentiation that we expect to see between distantly diverged strains was a reasonable one. By contrast, the mice used in this study were all inoculated with the same individual’s stool sample and were therefore not independent. As a result, we could not accurately quantify the number of differences that are expected between two unrelated hosts, which is crucial in determining whether the amount of intermediate frequency variants within a sample is 10% or less of the number of between-host SNV differences for a particular species, making it impossible to detect QP strains using this dataset alone. To overcome this issue, we calculated the expected number of between-host SNV differences for each focal species based on the aforementioned dataset used in Garud et al., 2019^20^.

### Inferring SNV frequency changes

To detect SNV frequency changes arising as a result of evolution, pairs of QP strains were compared. A SNV frequency change is defined as an allele with low frequency in one sample (*f* ≤ 0.2) and high frequency in the other sample (*f* ≥ 0.8). SNV differences were only considered at loci that had a coverage *D* ≥ 20 in both samples being compared. An extreme allele frequency change of this magnitude and this minimum depth is statistically unlikely to arise between QP strains due to sampling error or drift given large population sizes^20,33^. Pairs of samples harboring ≤ 50 SNV differences were considered to have the same inoculum strain evolving over short timescales (e.g., the eight-week timescale considered in this study), as larger amounts of SNV differences, such as *O*(10^13^) or greater are inconsistent with within-host rates of diversification (Garud et al., 2019). As a consequence, pairs of QP strains with > 50 SNV differences were excluded from this evolutionary analysis. For more information, see the Supplementary Information for Garud et al., 2019^20^.

### Bootstrapping confidence intervals for SNV difference rates

To measure whether there were significant differences in the number of SNV differences occurring between samples within hosts, between hosts, and between the inoculum and mouse hosts, we employed a bootstrap approach to compute confidence intervals for the rate of SNV difference between QP pairs of samples with the same dominant strain. In 1,000 bootstrap iterations, we sampled 100 QP sample pairs (not necessarily of the same species) with replacement from each comparison category: within host, between host, and between inoculum and host. For the within-cage comparison category, we only considered QP pairs from different hosts. We computed the actual rate of SNV differences for a particular comparison category as the total number of SNVs displaying extreme allele frequency differences across all sampled QP pairs divided by the total number of high coverage loci across all sampled QP pairs. We calculated 95% confidence intervals using the cumulative distribution function (CDF) of the overall SNV change difference rate in each comparison category generated from the 1,000 bootstrap iterations, taking the 2.5 and 97.5 percentile as the upper and lower bound, respectively (Figure 5B).

## Supplementary Material

Supplement 1

Supplement 2

Supplement 3

## Data Availability

Raw metagenomics sequencing reads are available on NCBI under BioProject [project accession number will be made available upon publication]. All necessary metadata, as well as the source code for the sequencing pipeline, downstream analyses, and figure generation, are available at GitHub (https://github.com/garudlab/Wasney-Briscoe/).
