# Supplementary material for: Uniform bacterial genetic diversity along the guts of mice inoculated with human stool
Source: bioRxiv. 2025 Jan 29:2025.01.28.635365. Preprint. [Version 1] doi: 10.1101/2025.01.28.635365 (PMC11838389; doi:10.1101/2025.01.28.635365)
Supplement: Supplement 1 [file media-1.pdf]

Supplementary Materials

Table of Contents

List of tables

Supplementary Table 1. Sequencing reads per sample
Supplementary Table 2. π estimates
Supplementary Table 3. Allele frequencies of SNVs experiencing extreme frequency changes

List of Figures

Supplementary Figure 1**.** Principal coordinate analysis (PCoA)
Supplementary Figure 2. Species considered for each analysis
Supplementary Figure 3. Relative strain frequency along the gut
Supplementary Figure 4. Evolutionary changes along the gut
Supplementary Figure 5. Inferring strain frequency of *Bacteroides vulgatus* strains
Supplementary Figure 6. Incorrect strain frequency inference of *Bacteroides uniformis* strains.

Tables

**Subject**	**Tissue**	**Paired-end reads per sample**
Mouse 1	Duodenum	34,126,263
	Jejunum	34,729,946
	Ileum	45,325,529
	Cecum	33,057,592
	Colon	32,374,899
Mouse 2	Duodenum	30,911,961
	Jejunum	34,871,836
	Ileum	34,789,365

	Cecum	32,920,833
	Colon	29,564,608
Mouse 3	Duodenum	36,008,731
	Jejunum	44,174,370
	Ileum	31,686,124
	Cecum	32,002,846
	Colon	30,546,791
Mouse 4	Duodenum	23,398,036
	Jejunum	30,787,783
	Ileum	33,305,802
	Cecum	29,389,586
	Colon	32,240,930
Mouse 5	Duodenum	33,612,764
	Jejunum	33,446,740
	Ileum	39,828,361
	Cecum	31,864,259
	Colon	30,393,912
Mouse 6	Duodenum	23,235,658
	Jejunum	31,781,992
	Ileum	51,606,729
	Cecum	34,283,821
	Colon	33,540,305
Mouse 7	Duodenum	66,099,259
	Jejunum	63,707,835

	Ileum	63,179,583
	Cecum	62,318,310
	Colon	68,214,936
Mouse 8	Duodenum	64,230,906
	Jejunum	107,687,261
	Ileum	130,428,298
	Cecum	109,849,940
	Colon	136,145,957
Inoculum		119,268,305
**Median of all samples**		33,948,292
**Median of all mouse samples**		33,612,764

Supplementary table 1. Sequencing reads per sample. The total number of metagenomic reads is shown for all samples. The median sequencing depth across all samples is given both including and excluding the inoculum.

[Supplementary table 2 attached as csv file]

Supplementary table 2. π **estimates.** π, a measure of nucleotide diversity, was estimated in all mouse samples and the inoculum for species that had at least 500,000 loci that had a coverage ≥ 4 in a given sample.

[Supplementary table 3 attached as csv file]

Supplementary table 3. Allele frequencies of SNVs experiencing extreme frequency changes. Allele frequencies of all SNVs detected as having gone from an allele frequency of $f \leq 0.2$ to $f \geq 0.8$ between any pair of samples. SNVs are annotated according to "contig", position in the reference genome ("site_pos"), PATRIC ID ("gene"), gene annotations lifted from the PATRIC database ("gene_description"), "codon_degeneracy" (where 1D indicates that the site is 1-fold degenerate such that any nucleotide difference will result in an amino acid change, whereas 4D indicates that the site is 4-fold degenerate such that any nucleotide difference will not result in an amino acid change). Subsequent columns indicate the sample in which the allele

frequencies are calculated. An allele frequency value of NA indicates that the nucleotide site did not meet the minimum read coverage requirement of 20 reads or more in that sample.

Supplementary Figures

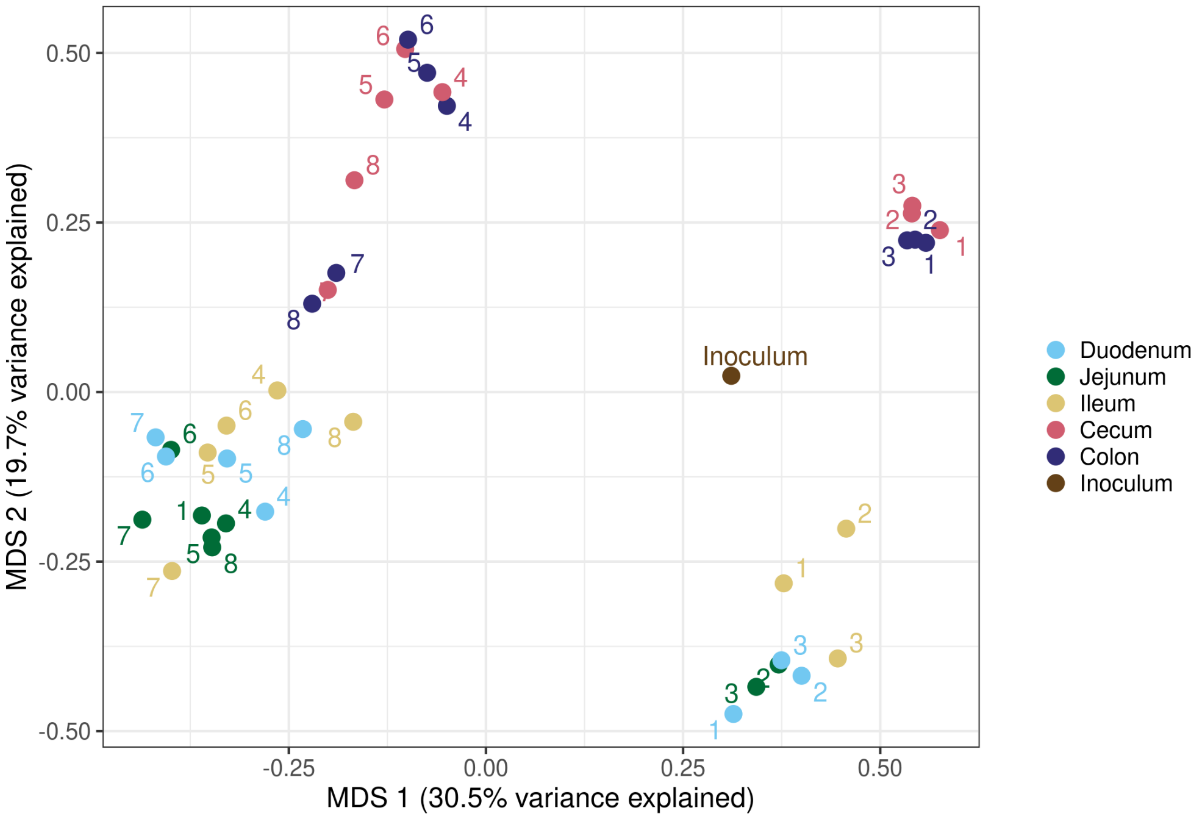

Supplementary Figure 1. Principal coordinate analysis (PCoA) Distances computed using beta diversity (Bray-Curtis Dissimilarity Index) values calculated from relative bacterial species abundances. Samples cluster by gut region, indicated by color. Each point is labeled with the mouse identity (1–8) or inoculum.

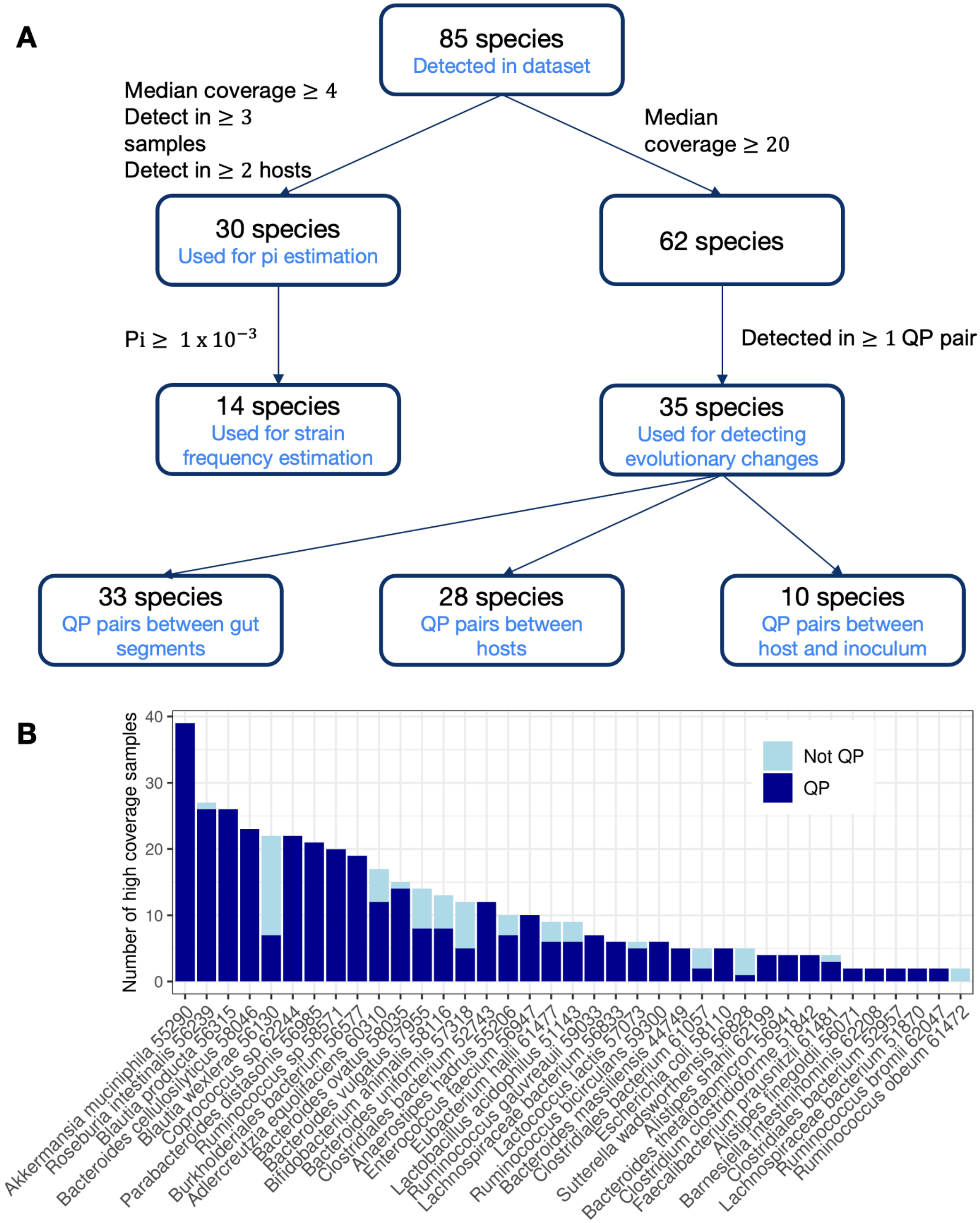

Supplementary Figure 2. Species considered for each analysis (A) Species requirements for assessing within-species diversity with pi, inferring strain frequencies, and assessing evolutionary changes within hosts, between hosts and between inoculum and host. **(B)** Number

of QP and non-QP pairs for all species in the dataset that have at least two high coverage samples (i.e., median coverage ≥ 20).

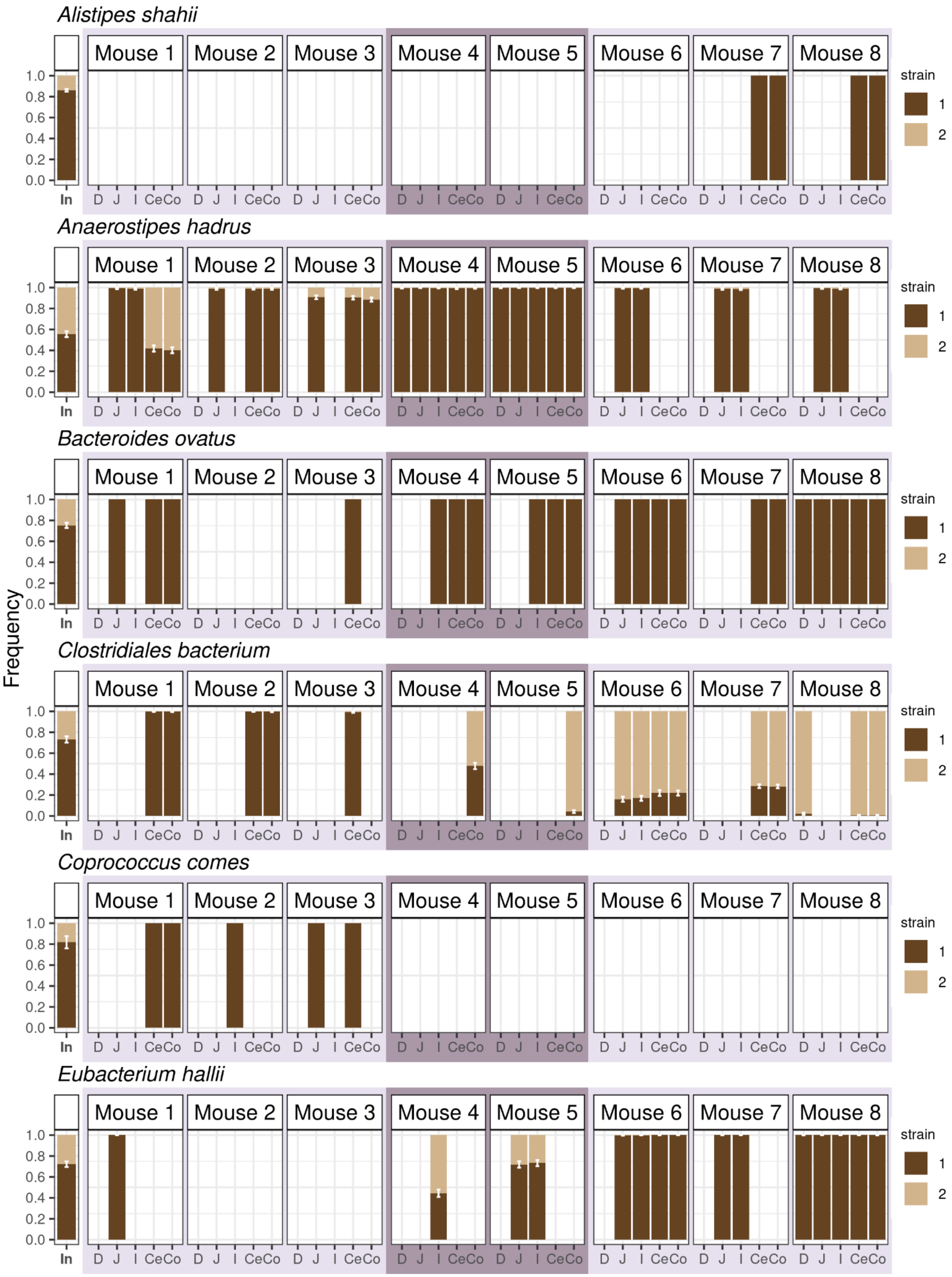

Alistipes shahii
Anaerostipes hadrus
Bacteroides ovatus
Clostridiales bacterium
Coprococcus comes
Eubacterium hallii
Mouse 1
Mouse 2
Mouse 3
Mouse 4
Mouse 5
Mouse 6
Mouse 7
Mouse 8
Frequency
1.0
0.8
0.6
0.4
0.2
0.0
In
D J I CeCo
strain
1
2

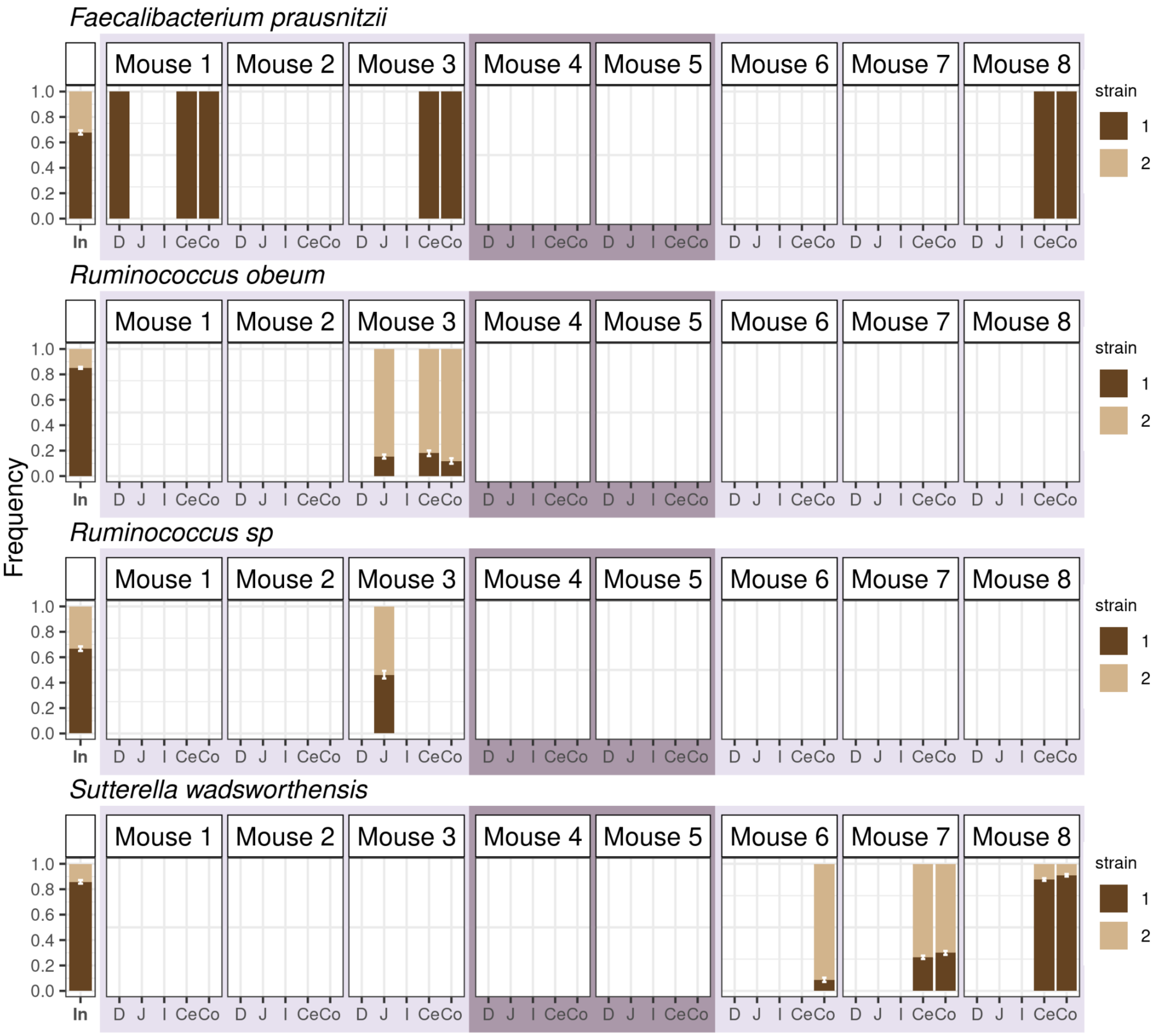

Supplementary Figure 3. Relative strain frequency along the gut. Strain frequency of oligo-colonizing strains was inferred across all samples for the 11 species that had $\pi \geq 1 \times 10^{-3}$ in the inoculum. Strain frequencies of the four species not shown here can be found in Figure 4 of the main text. Strain frequency is indicated on the y-axis, with error bars representing the 95% confidence intervals for the inferred strain frequency (**Methods**).

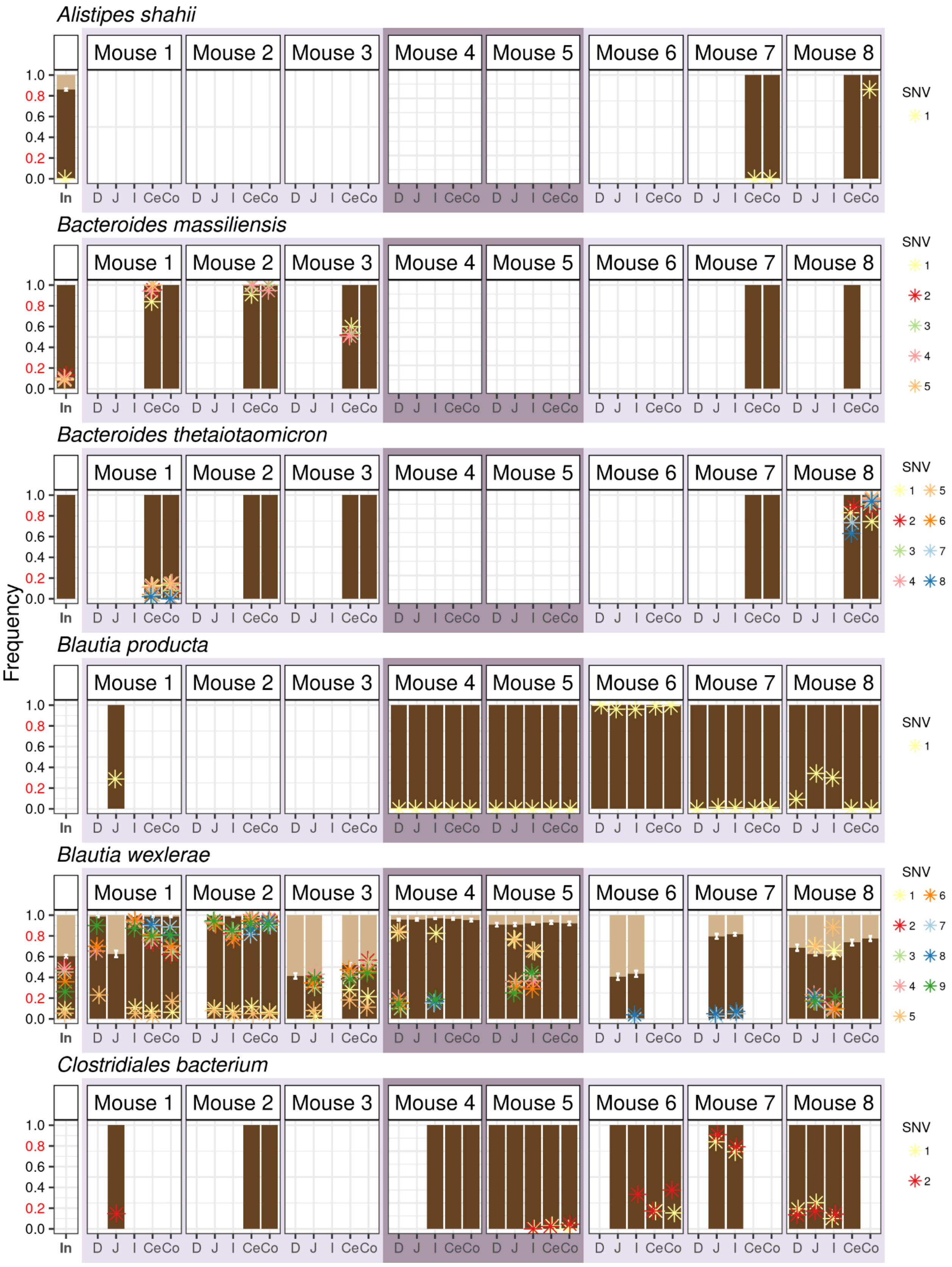

Alistipes shahii
Bacteroides massiliensis
Bacteroides thetaiotaomicron
Blautia producta
Blautia wexlerae
Clostridiales bacterium
Mouse 1
Mouse 2
Mouse 3
Mouse 4
Mouse 5
Mouse 6
Mouse 7
Mouse 8
Frequency
1.0
0.8
0.6
0.4
0.2
0.0
In
D J I CeCo
SNV
1 2 3 4 5 6 7 8 9

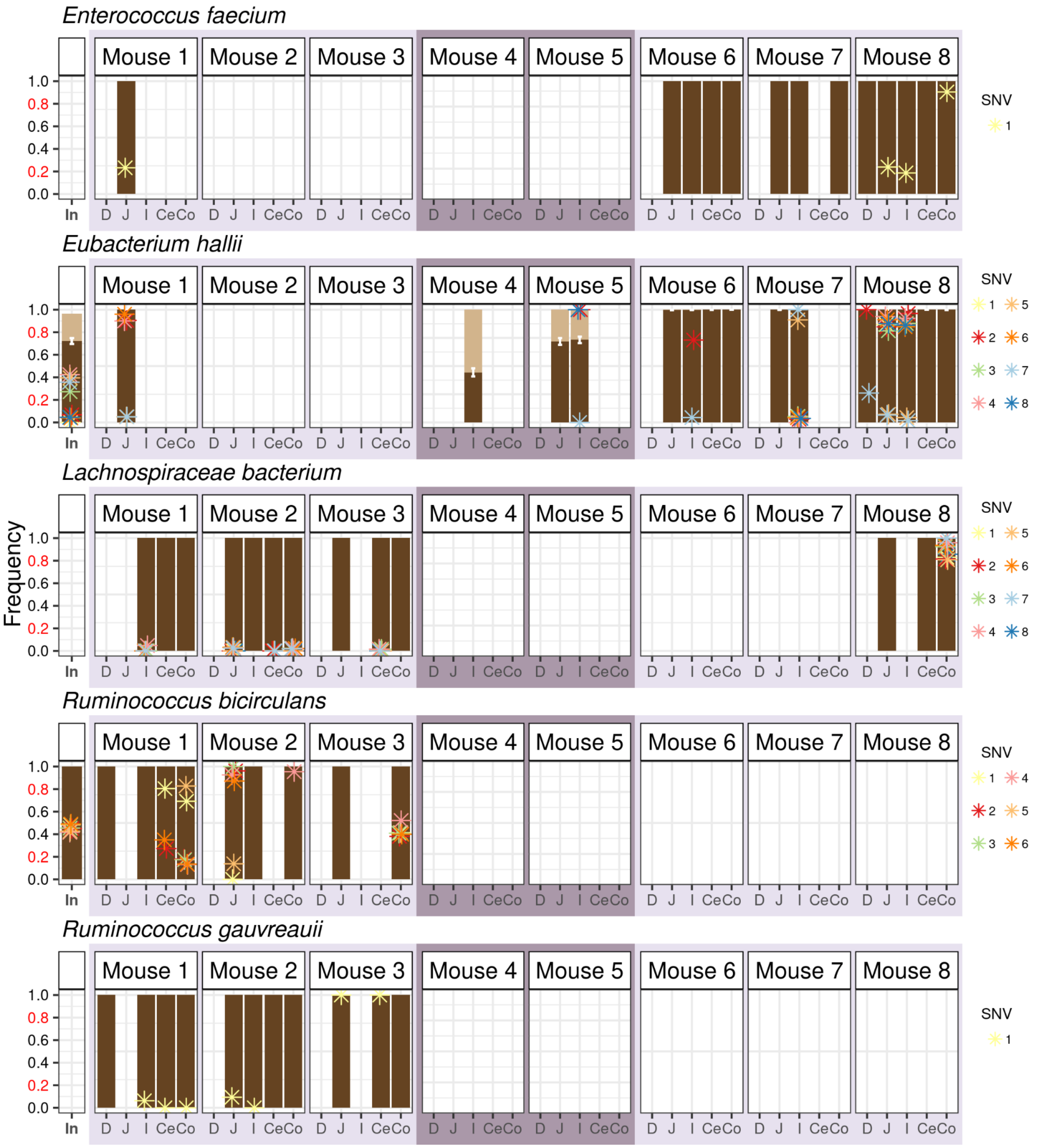

Supplementary Figure 4. Evolutionary changes along the gut. SNVs in 13 species underwent extreme allele frequency changes (i.e., $f \leq 0.2$ to $f \geq 0.8$) between pairs of QP samples. Allele frequencies for these SNVs were calculated in all samples for which the loci of interest had a coverage $D \geq 20$. Asterisks represent the allele frequency of a given SNV, with each SNV within a species having its own unique color. Samples lack asterisks for particular SNV when those loci do not have adequate coverage to infer allele frequency. When multiple strains of the same species are present, frequencies of co-colonizing strains are represented as dark and light shades of brown (see **Supplementary Figure 3** for legend).

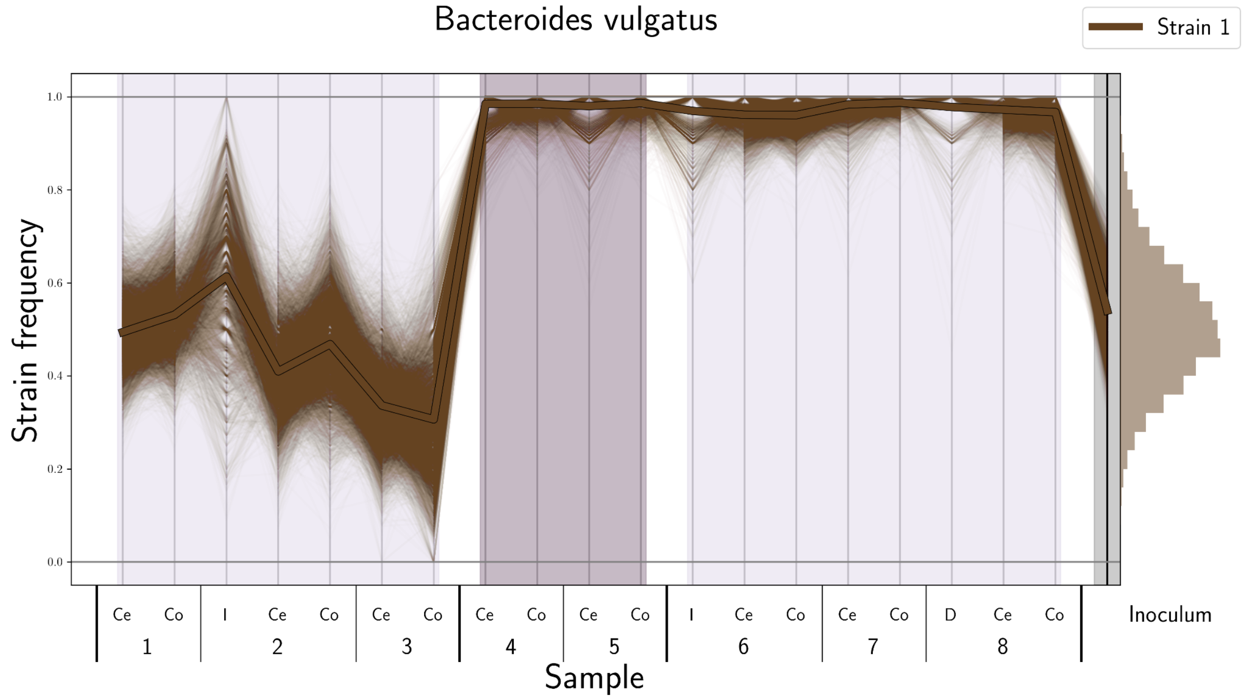

Supplementary Figure 5. Inferring strain frequency of *Bacteroides vulgatus* strains. Strain frequencies were inferred for *B. vulgatus* (and other species) by clustering loci into large groups of SNVs that display highly correlated allele frequencies (thin brown lines) across samples (indicated by labels on the x axis; “I” indicates ileum, “Ce” indicates cecum, and “Co” indicates colon; numbers indicate mouse identity). The strain frequency was inferred to be the mean of the inferred clusters (thick brown line). In the case of *B. vulgatus*, one cluster was inferred, distinguishing two strains. The histogram to the right of the plot represents the distribution of allele frequencies of clustered SNVs in the inoculum.

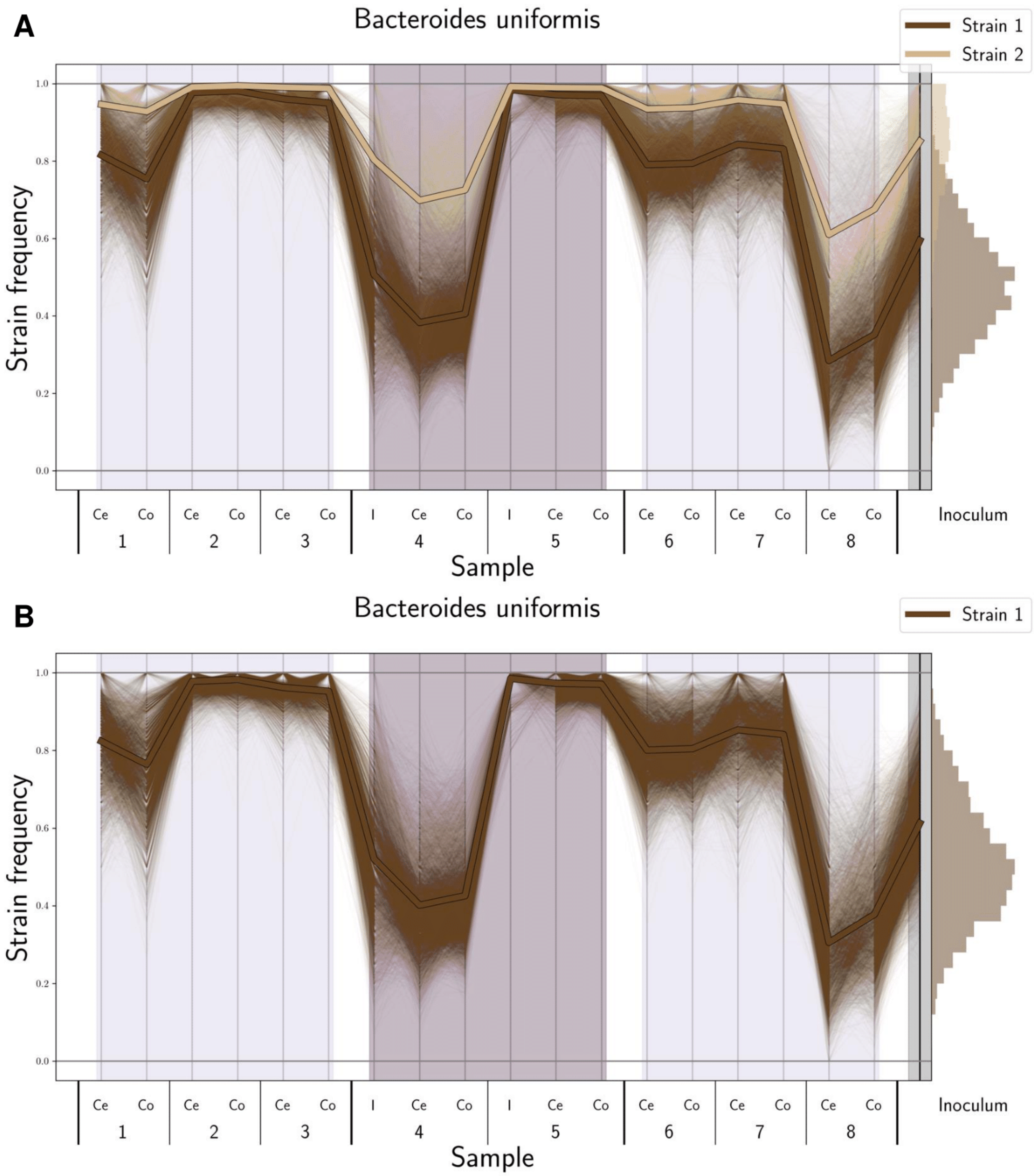

Supplementary Figure 6. Incorrect strain frequency inference of *Bacteroides uniformis* strains. (A) *B. uniformis* SNVs were clustered into two groups, despite representing a single group of correlated SNVs. **(B)** The two clusters were merged, and SNVs that had a distance $d \leq 3.5$ with 25% of other SNVs in the cluster were retained.
